# Adipokines in hereditary breast cancer patients and healthy relatives

**DOI:** 10.18632/oncotarget.21018

**Published:** 2017-09-18

**Authors:** Domenico Sambiasi, Simona De Summa, Maria Digennaro, Brunella Pilato, Angelo Paradiso, Stefania Tommasi

**Affiliations:** ^1^ Experimental Medical Oncology, IRCCS Istituto Tumori “Giovanni Paolo II”, 70124 Bari, Italy; ^2^ Molecular Genetic Laboratory, IRCCS Istituto Tumori “Giovanni Paolo II”, 70124 Bari, Italy

**Keywords:** adipokines, BRCA, breast cancer, BMI, hereditary

## Abstract

**Background:**

The role of adipocytokines and ghrelin in hereditary breast cancer syndrome (HBCS) has never been tested.

**Results:**

No significant differences in leptin, adiponectin and ghrelin plasma levels between cancer patients and healthy subjects was observed. Conversely, an higher level of adiponectin was shown in healthy subjects with BRCA 1/2 gene mutation vs those without (*p* < 0.03). Logistic regression analysis demonstrated that Adiponectin plasma level (OR 0.26; 95% CI:0.007–0.81; *p* < 0.02) and age (OR 5.51; 95% CI:1.78–19.71; *p* < 0.004) were the only factors independently associated with BMI; furthermore, Leptin plasma level (OR 0.23; 95% CI:0.06–0.76; *p* < 0.01) and age (OR 0.05; 95% CI:0.05–0.61; *p* < 0.007) resulted the only factors significantly associated with breast cancer.

**Materials and Methods:**

We analyzed blood plasma expression of leptin, adiponectin and ghrelin using Bio-Plex platform in 25 breast cancer patients with HBCS and in 38 healthy relatives. BRCA 1/2 gene status (presence of pathogenic mutations by direct molecular sequencing), clinical-pathological characteristics and Body Mass Index (BMI) of each subject were recorded.

**Conclusions:**

Adiponectin confirms to be associated with BMI also in subjects with HBCS. Leptin plasma level seems a direct and independent biomarker of a breast cancer risk. A validation of Leptin as a circulating biomarker of breast cancer development in larger series of HBCS subjects is needed.

## INTRODUCTION

Breast cancer is the female malignant neoplasia with the highest incidence in the industrialized world [1]. However, despite many undeniable therapeutic success obtained thanks to prevention and early diagnosis, breast cancer still remains, a major cause of death for woman [2]. Several risk factors for this disease have been reported as age, parity, breast density, prior thoracic radiotherapy and family history of breast cancer [3].

The risk for sporadic breast cancer is associated with obesity through a number of different mechanisms including insulin resistance, induction of a metabolic syndrome, insulin-like growth factor modulation, chronic low-grade inflammation [4–8]; In addition, circulating levels of estrogens have been demonstrated to be strongly and linearly related to adiposity in postmenopausal breast cancer patients [4] and, even more interesting, body mass index (BMI) to be positively related with plasma concentration of adipokines, including leptin, adiponectin, ghrelin, tumor necrosis factor (TNF)-α, and interleukin-6 (IL-6) [9].

All these information have been supported by a recent meta-analysis highlighting that obesity contributes to increase breast cancer risk in a nonlinear dose-response manner in postmenopausal women and that body weight control may be a crucial process to reduce breast cancer susceptibility [8].

Adipokines are directly involved in body weight regulation and the pathogenesis of various disease through different mechanisms such as inflammatory response promotion, induction of cardiovascular diseases, inducing diabetes [10–11]; their role in these physio-pathological processes suggested also their utilization as attractive therapeutic targets [12–13].

Adiponectin, is secreted exclusively from adipose tissue with higher serum level associated with female gender and inversely related to body weight [9–10]. Several reports have indicated association between low adiponectin levels and elevated risk of breast, endometrial, and gastric cancers [14–16].

Leptin, another member of the adipocytokines family, is produced mainly by differentiated adipocytes and is active at central nervous system level to suppress food intake need and stimulate energy expenditure [17, 18]. Leptin levels were also reported to be lower in gastrointestinal and pancreatic cancer patients [19–21] but higher in breast and gynecologic cancer patients [21].

The major source of ghrelin is the stomach, where it is synthesized in distinct endocrine cell type, known as the X/A-like cells [22]. The peptide is a powerful inducer of growth hormone (GH) release, acting at the pituitary and hypothalamic levels [23]. Ghrelin play a role in the regulation of cell proliferation in different tissues and tumors, with high levels in prostate, lung, gastrointestinal, and breast cancers [24].

Little is known about relationships between BRCA and adipokines cell pathways. Indirect evidence for a relationship come from some controlled clinical trials analyzing the role of physical activity and body weight in BRCA1/2 mutation carriers; Bordelau [25] demonstrated that risk for breast cancer is lower in BRCA1/2 carriers physically active and with lower weight; moreover, Kotsopoulos [26] showed in a case control study that weight loss and avoiding weight gain both reduced breast cancer risk among BRCA1/2 carriers.

Taken together, all these evidences support the hypothesis of adipokines as risk factor in particular for breast cancer and of their involvement in familial disorders based on specific genetic alterations like obesity, metabolic syndrome, diabetes. However, the intriguing hypothesis that those factors could play a peculiar role also in oncogenetic familial disorder like the hereditary breast cancer syndrome has never been tested before.

In the present study, we analyzed by a multiplex ELISA plasma-blood assay, the expression of leptin, adiponectin and ghrelin in breast cancer patients and their healthy relatives belonging to families with Hereditary Breast Cancer Syndrome and with known BRCA1/2 gene molecular status.

## RESULTS

The baseline characteristics of cases and controls are summarized in Table 1. The BMI mean value and mean age resulted higher in cancer patients than in control subjects (27.5 vs 24.8 Kg/m^2^, *p* < 0.000; 44.2 vs 36.6 years, *p* < 0.01, respectively). To exclude the hypothesis that the different mean age of patient and control groups could conditionate the results, we analyzed the expression of our biomarkers, by Pearson test, with respect to age in healthy people confirming that the level of biomarkers was independent from age of subjects (data not shown).

**Table 1:** Baseline clinical characteristics of HEREDITARY cancer patients and their healthy relatives

Characteristics	Cancer patients (*n* = 25)	Healthy (*n* = 38)	*P* value
Age (years Mean ± SD)	44.20 ± 6.14	36.63 ± 8.54	0.011
Body Mass Index, BMI (Kg/mq, Mean + SD)	27.56 ± 4.94	24.77 ± 3.0	0.01
No. patients (%)			
BMI Low (< 25)	11 (44.0)	19 (52.7)^a^	
			0.34
BMI High (> 25)	14 (56.0)	17 (47.2)^b^	
No. patients (%)			
BRCA mut carriers	11 (44.0)	17 (44.7)	
			0.58
BRCA wt carriers	14 (56.0)	21 (55.3)	
Adiponectina μg/ml	27.83 ± 18.8	32.58 ± 16.4	0.306
Leptin ng/ml	18.20 ± 19.8	11.68 ± 11.5	0.146
Ghrelin pg/ml	991.74 ± 531.0	1153.94 ± 582.8	0.259

Furthermore, no significant differences in leptin, adiponectin and ghrelin plasma levels between cancer patients and healthy control was observed.

**Figure 1:**
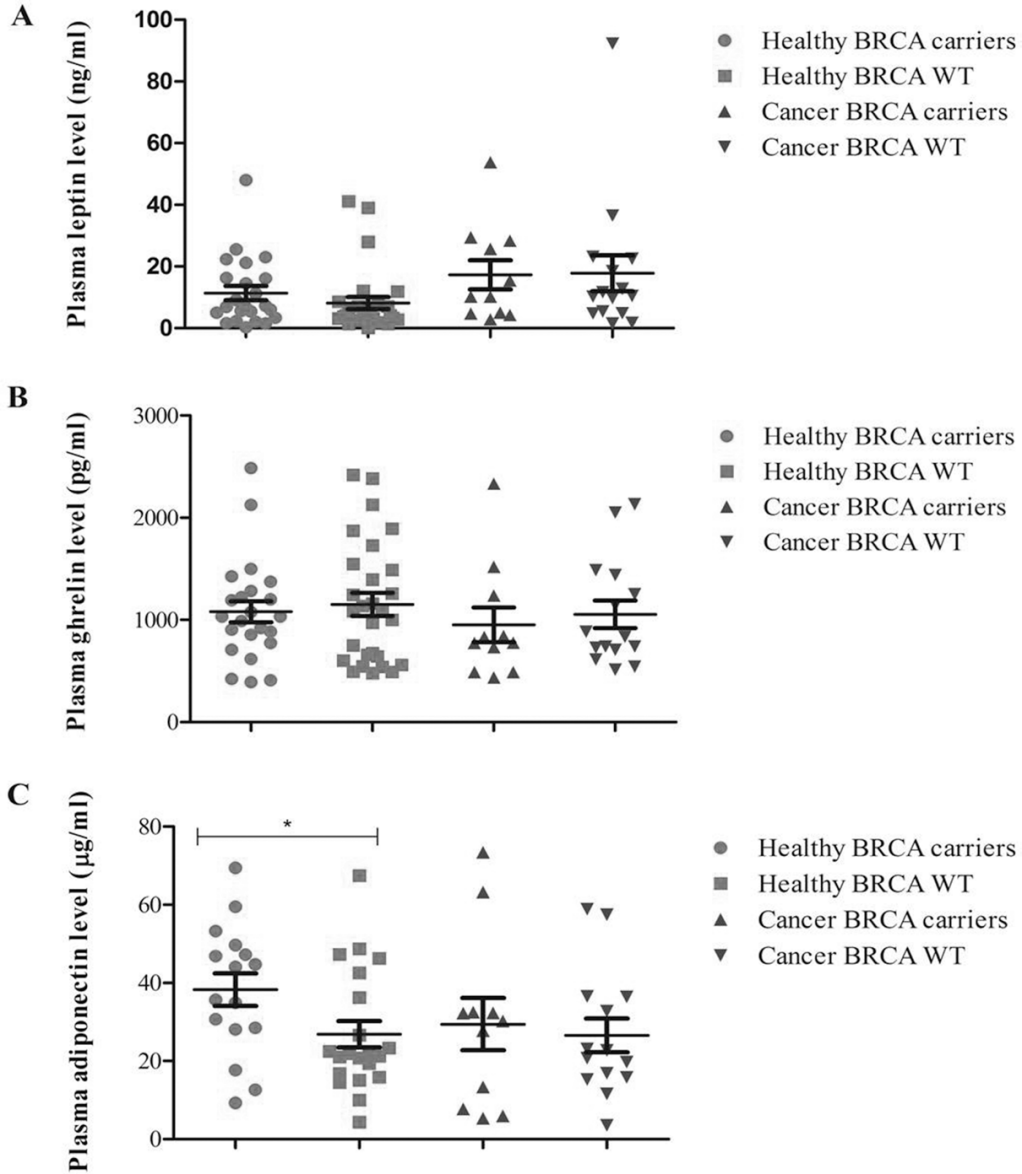
Plasma expression profile of the analytes with respect to BRCA status in breast cancer patients and healthy relatives

When the biomarker plasma expression was evaluated with respect to BRCA mutation status in patients and healthy subjects separately (Figure 1C), an higher level of adiponectin was shown in BRCA mutated than in BRCA wild type healthy subjects (38.3 vs 26.8 μg/ml, respectively, *p <* 0.038); no further significant differences were found for leptin and ghrelin with respect to BRCA status and disease status (Figure 1B–1C).

**Figure 2:**
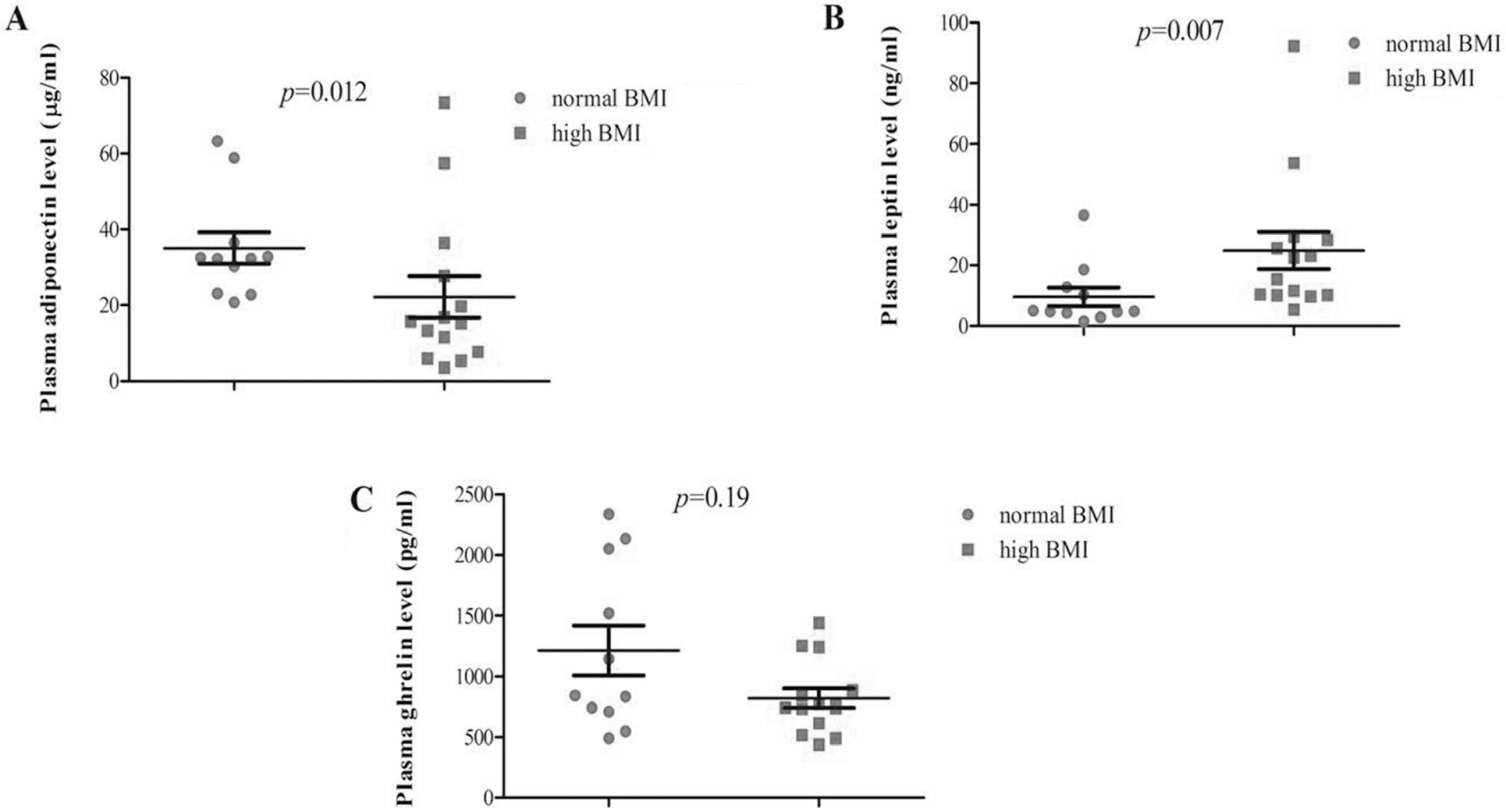
Plasma analyte levels according to BMI categories (normal vs high) in breast cancer patients

Furthermore, biomarker expression was analyzed with respect to BMI: a significant association was found with Leptin levels (24.9 vs 9.6 ng/ml in high and low BMI, respectively; *p* < 0.007, Figure 2B) while the association resulted inverse with Adiponectin (22.2 vs 35.0 μg/ml, in high and low BMI, respectively; *p* < 0.012, Figure 2A). The relationship with BMI was also analyzed by a logistic regression model demonstrating that Adiponectin plasma level (OR 0.26; 95% CI:0.007–0.81; *p* < 0.02) and age (OR 5.51; 95% CI:1.78–19.71, *p* < 0.004) were the only factors significantly retained in the final model while BRCA status, disease status, Leptin and Ghrelin expressions did not (Table 2 ). Finally, we conducted a logistic regression analysis with disease status (breast cancer vs healthy status) as dependent variable demonstrating that Leptin serum level (OR 0.23; 95% CI:0.06–0.76; *p* < 0.01) and age (OR 0.05; 95% CI:0.05–0.61 *p* < 0.007) were the only factors significantly retained in the final model while BRCA status, Adiponectin and Ghrelin did not (Table 3).

**Table 2:** Logistic regression analysis with BMI as dependent variable and age, BRCA status, and biomarker level included in the model^*^

	OR (95%CI)	*p*-value
**Adiponectin** Low vs High level	0.26 (0.07÷0.81)	0.02
**Age** Old vs Young	5.51(1.78÷19.71)	0.004

^*^BMI: cut-off value > 25;

Age: cut-off (median value of the overall series); BRCA status: mutated vs wild type;

Biomarker expression: cut-off median value of the series.

**Table 3:** Logistic regression analysis with Disease status (Breast cancer vs Healthy) as dependent variable and age, BRCA status, biomarker expression included in the model^*^

	OR (95%CI)	*p*-value
Leptin Low vs High level	0.23 (0.06 ÷ 0.76)	0.01
Age Young vs Old	0.05 (0.05 ÷ 0.61)	0.007

^*^(for cut-off values, see Table 2 legend)

## DISCUSSION

Adipokines exert several physiological effects involving different cell pathways and producing various biological results such as modulation of inflammatory responses, functionality of vascular endothelial cell adhesion molecules, characteristics of extracellular matrix components [11] but till now there is no information on adipose-derived secreted factors, i.e. adipokines, and cellular BRCA mediated pathways.

For the first time, BMI and adipokynes plasma-blood levels were analyzed in a series of subjects belonging to hereditary breast cancer families. The rationale of our study was based on the clear evidence these factors play in genesis and progression of several cancers but also, even more important, on their involvement in other hereditary syndromes like diabetes [27].

Preliminarily, we confirmed also in this series of subjects with hereditary breast cancer features, the difference in BMI and age characteristics of breast cancer patients with respect to healthy people, relationship that we already showed in a large population of subjects without hereditary features [28]. This information, already reported by Bordeleau [25], confirms that obesity is an important risk factor for hereditary breast cancer too. Even more important, we confirmed in this peculiar series of subjects that Adiponectin serum levels inversely relates with body weight. In conclusion, after several reports (7–8) indicating an association between low adiponectin level and BMI in sporadic breast cancer, we here demonstrate by multiple logistic regression analysis that this is the case also for hereditary breast cancer and independently from other main clinical factors. We suggest that Adiponectin could be utilized as a surrogate biomarker for BMI evaluation but we have to stress that the kinetics of this serum marker with respect to time of development of the obesity is still unknown. Does this plasma biomarker increase preced the clinical appearance of the obese phenotype, eventually?

One more interesting information coming from our study concerns Leptin; Leptin serum levels seem directly and independently from other factors related to the presence of breast cancer. In fact, in our experience, a low Leptin level is associated with an healthy status (OR:0.23; 95% CI 0.06–0.76); however, also in our experience, the strongest predictive factor for breast cancer development resulted an older age. Can we look at Leptin as a potential clinical biomarker for the development of breast cancer in subjects belonging to hereditary breast cancer families? This question is now under study in our Institute in a successive large series of subjects with same familial characteristics and aimed to confirm the effects of Adjuvant Endocrine treatment on serum Leptin, Serum Adiponectin and body composition of patients with breast cancer [29].

The last result concerns the relationships between adipokines and BRCA status. In general, in our series of patients we were not able to confirm a clear relationships between those factors thus supporting the idea that biomolecular pathways associated with BRCA genes (DNA repair) and fat hormone metabolism could be not directly related. Further studies on larger series of patients are requested to confirm this hypothesis.

## MATERIALS AND METHODS

### Patient population

From January 2010, a consecutive series of subjects having access to Centro Studi Tumori Eredo-Familiari of Istituto Tumori of Bari for breast–ovarian hereditary cancer syndrome was selected to be enrolled to the present study. In specific, 25 breast cancer patients and 38 healthy relatives who met clinical criteria for genetic counseling and analysis of BRCA mutation status according to procedure and criteria already published [30] entered the present study. 11/25 breast cancer patients and 17/38 healthy relatives belonging to families with hereditary breast cancer syndrome resulted BRCA mutation carriers. For each patient and relatives involved in this study the following information was available: i) exact BRCA status of the family, ii) date of genetic testing, iii) age at sample collection, iv) Body Mass Index (BMI). For 25 breast cancer patients surgical, radiation therapy, systemic adjuvant treatment, and pathological informations were also recorded. The study was approved by IAB of our Cancer Institute in May/2014. All subjects candidate to enter the study, signed a specific consent form.

Plasma samples for ghrelin, adiponectin and leptin levels determination by protein array Bio-Plex platform were collected at the time of genetic counseling for suspected hereditary breast-ovarian cancer syndrome. At the time of blood collection, the patients were free from any type of cancer treatment (surgery, radiotherapy, hormone-therapy, chemotherapy) since at least 3 months.

### Plasma analysis using bio-plex suspension array system

Bio-Plex Suspension Array System produced by Bio-rad Laboratories Inc, based on a Microplate-ELISA bioassay system was utilized for analysis of each biomarker as previously reported [31]. The Human Diabetes assay Panel was utilized for Adipokine, leptin, ghrelin analysis [31]. Plasma-blood adiponectin, leptin, and ghrelin assays were performed with an intra-assay CV of 4%, 3%, and 4%, respectively (Bio-Rad Laboratories, Inc, Berkeley, CA, USA). Analysis were purchased from BioClarma-Research and Molecular Diagnostics , Turin.

### Statistical analysis

#### Univariate analysis was preliminarly performed for each of Biomarkers

Comparison between independent groups was performed by *t*-test. The difference in percentage between independent groups and the relationship between different variables were analyzed by means of Pearson's Chi-square test. Multiple Regression logistic analyses, with backward procedure and AIC stepwise selection, were performed whit dependent variable BMI (low vs high BMI with cut-off BMI >= 25) and Health status (cancer vs healthy status) including as independent variables, age (cut off median age of the series), BRCA status (mutated vs wild-type), and analytes levels (categorized in low vs high level according to median value of the series). All statistical analyses were carried out using SPSS statistical software [32].

## Abbreviations

HBCS: hereditary breast cancer syndrome
BMI: Body Mass Index
RT: radiotherapy
IGF: insulin-like growth factor
TNF-α: tumor necrosis factor
IL-6: interleukin-6
GH: growth hormone
